# T-cell autonomous death induced by regeneration of inert glucocorticoid metabolites

**DOI:** 10.1038/cddis.2017.344

**Published:** 2017-07-20

**Authors:** Lourdes Rocamora-Reverte, Holger M Reichardt, Andreas Villunger, GJan Wiegers

**Affiliations:** 1 Biocenter, Division of Developmental Immunology, Medical University, Innsbruck, Austria; 2 Institute for Cellular and Molecular Immunology, University Medical Center Göttingen, Göttingen, Germany; 3 Tyrolean Cancer Research Institute, Innsbruck, Austria

## Abstract

Glucocorticoids (GC) are essential regulators of T-cell development and function. Activation of the immune system increases systemic adrenal-derived GC levels which downregulate immune activity as part of a negative feedback control system. Increasing evidence shows, however, that GC can also be derived from extra-adrenal sources such as the thymus or intestine, thus providing local control of GC-mediated effects. The thymus reportedly produces GC, but whether thymic epithelial cells or thymocytes produce GC acting either in an autocrine or paracrine fashion is not clear. We studied the expression of two main enzymes involved in *de novo* GC synthesis, CYP11A1 and CYP11B1, as well as the expression and activity of HSD11B1, an enzyme catalyzing interconversion of inert GC metabolites with active GC. While we found no evidence of *de novo* GC synthesis in both thymocytes and peripheral T cells, abundant regeneration of GC from the inactive metabolite 11-dehydrocorticosterone was detectable. Irrespective of their maturation stage, T cells that produced GC in this manner undergo autonomous cell death as this was blocked when glucocorticoid receptor-deficient T cells were treated with GC metabolites. These results indicate that both immature and mature T cells possess the capacity to undergo apoptosis in response to intrinsically generated GC. Consequently, positive selection of thymocytes, as well as survival of peripheral T cells may depend on TCR-induced escape of otherwise HSD11B1-driven autonomous T-cell death.

Glucocorticoids (GC) are steroid hormones primarily produced in the adrenal cortex in response to emotional, physical and immunological stress. Corticosterone, the predominant GC in mice, and its human homolog cortisol, have numerous effects on diverse processes such as metabolic activity, immune function and behavior.^1^ GC bind to their receptor, the glucocorticoid receptor (GR), which reduces the expression of many pro-inflammatory cytokines and it is generally assumed that this explains the potent anti-inflammatory and immunosuppressive properties of GC.^2^

The thymus is the key immunological organ for the maturation of T cells in mammals. Elevation of GC due to chronic stress or experimental administration causes involution of the thymus due to the fact that GC are strong inducers of apoptosis in thymocytes and have a critical role in their development and function. Immature double-negative (DN) thymocytes (CD4−CD8−) proliferate and differentiate in the thymus to generate double-positive (DP) CD4+CD8+ cells. Most of these DP cells undergo apoptosis; the surviving differentiate into single-positive (SP) CD4+ or CD8+ cells that migrate to peripheral lymphoid tissues.^3, 4^ Positive selection of developing thymocytes for progression from the DP to the SP stage requires low to moderate avidity TCR-mediated interactions with self-peptide/MHC ligands.^5, 6^ GC have been proposed to be essential for the selection of immunocompetent T cells.^7^ The mutual antagonism hypothesis proposes that a quantitative balance between TCR and GR signaling determines the fate of a developing thymocyte. GC thereby promote positive selection by antagonizing negative selection signals.^8, 9, 10, 11^ In contrast, TCR signaling increasingly reverses GC-induced apoptosis^12^ as thymocyte development progresses.^13^

While the main source of GC are the adrenals, evidence accumulated over the last two decades that GC are also *de novo* synthesized in other organs including the brain, intestinal tract, skin and thymus (both epithelial and immune cells).^14, 15^ Accordingly, these organs express the steroidogenic enzymes necessary for the synthesis of GC which apparently act in an autocrine or paracrine fashion.^3^ Overexpression of GR in the T-cell lineage leads to a reduced number of thymocytes in adrenalectomized mice, suggesting that non-adrenal-derived GC could exert a negative effect on thymocyte development.^16^ In the mouse thymus, however, there is considerable controversy about the cellular origin of GC synthesis. The presence of key enzymes for GC synthesis has been extensively described in thymic epithelial cells (TEC^10, 17^). On the other hand, some studies show the ability of thymocytes to synthesize GC.^18, 19^ Disagreement exists also on whether the expression of GC-synthesizing enzymes is dependent on T-cell activation status.^20, 21^

Of note, corticosterone can also be produced from the inactive metabolite 11-dehydrocorticosterone (11-DHC) via the reductase activity of HSD11B1, which is expressed by murine CD4+ and CD8+ lymphocytes.^22^ In thymocytes, *Hsd11b1* has been shown to be expressed at substantial levels^20^ and also to be functionally active.^23^ Along similar lines, we aimed to investigate the quantitative contribution of either *de novo* GC synthesis or conversion of 11-DHC to T-cell-derived corticosterone and tested whether this hormone displays intracrine activity. We performed a detailed analysis of the expression and activity of steroidogenic enzymes in mouse thymus and spleen, throughout T-cell development. Based on our findings, we can refute a significant role for CYP11B1 in GC synthesis, suggesting that neither thymocytes nor splenocytes synthesize significant amounts of GC *de novo.* In contrast, HSD11B1 converts inactive 11-DHC into active corticosterone that can induce subsequent thymocyte and T-cell death. Our findings highlight an underappreciated T-cell autonomous mechanism that can affect the T-cell selection process and contribute to the tolerizing effects and immune suppressive function of glucocorticoids.

## Results

### Expression analysis of glucocorticoid metabolic enzymes across T-cell development

**Figure 1:**
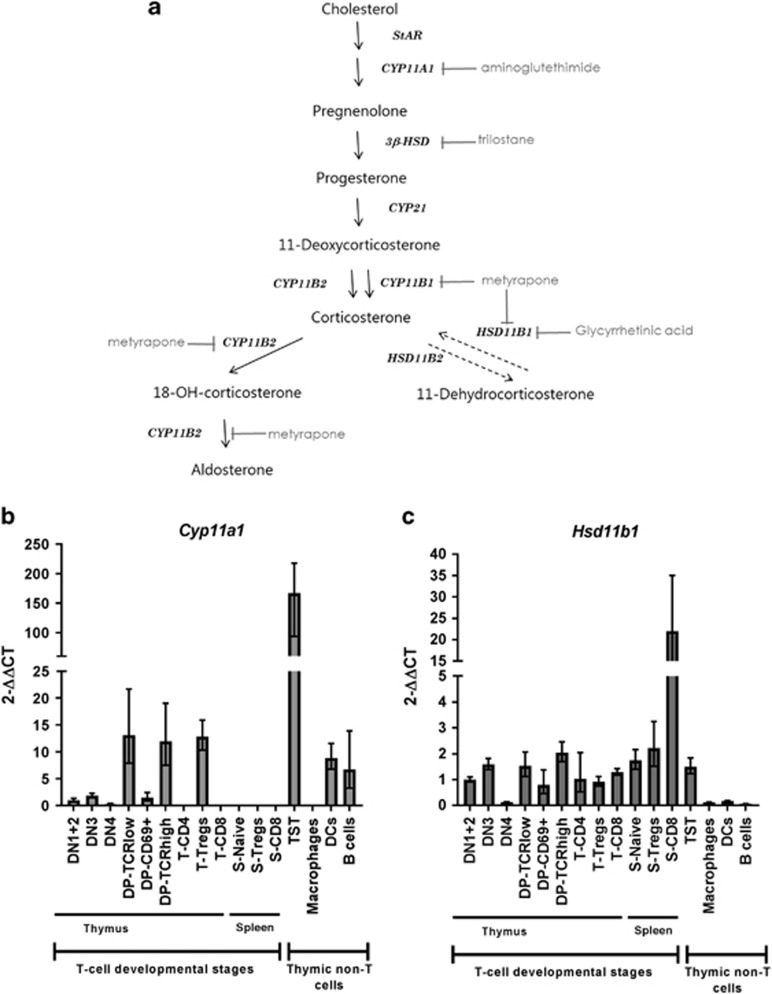
Differential gene expression of the main GC metabolic enzymes in thymus and spleen. (**a**) Schematic of the steps in glucocorticoid synthesis. Enzymes are shown in italics and inhibitors are depicted in gray. Solid arrows show *de novo* synthesis of GC; dashed arrows indicate activation/inactivation from inactive GC to the active form, respectively. Real-time qPCR analysis of *Cyp11a1* (**b**) and *Hsd11b1* (**c**) in different cell types from thymus and spleen of 5–12 weeks old mice. Expression level of each gene is expressed as 2^−^^ΔΔCt^ (referred to DN1+2 cells relative to *Actin*). DCs, dendritic cells; S, spleen; T, thymus; TST, enriched thymic stromal tissue

To date it is unclear which cell type(s) of the thymus are responsible for GC synthesis (Figure 1a), being TEC and/or thymocytes a matter of debate. To unravel the origin of thymus-derived GC production, we studied the expression of two critical steroidogenic enzymes in T cells at different developmental stages, from immature thymocytes to mature peripheral T cells in the spleen (Table 1). Expression levels of CYP11A1, the rate-limiting enzyme in steroidogenesis from cholesterol, and CYP11B1, which is responsible for the conversion of 11-deoxycorticosterone (11-DOC) into corticosterone, were quantified by qPCR. *Cyp11a1* was detectable at low levels at early developmental thymocytes stages (DN and DP cells) but its expression became undetectable when cells develop into more mature states. As shown in Figure 1b, we discriminated DP cells according to their maturation stage (TCR*β* expression level) and their activation/status (presence of CD69).^24^ We found no differences in TCRlow and TCRhigh DP cells but those which were positive for CD69 downregulated *Cyp11a1* expression.

Surprisingly, we did not detect *Cyp11b1* expression in any T-cell subset, either in thymus or in spleen (Table 1). Two different primer pairs for the detection of the encoding mRNA, validated on samples from the adrenal gland, failed to yield positive results in FACS-sorted thymocytes. Several reports have shown the expression of *Cyp11b1* by TEC, suggesting that at least this cell type can provide corticosterone for developing T cells. In a further attempt to detect *Cyp11b1* in the thymus, we used enriched thymic stromal tissue (TST) as previously described^20^ and checked for the expression of *Keratin-8 (Krt-8)*, an epithelial cell marker, to ensure the presence of TEC in the TST fraction (Supplementary Figure 1). As shown in Table 1, we also failed to detect *Cyp11b1* in enriched TST. Next, we isolated thymic B cells, dendritic cells (DCs) and macrophages and analyzed *Cyp11b1* expression, as well as whole thymus samples. In any case, *Cyp11b1* was undetectable (Table 1).

Yet, corticosterone can also be converted from the inactive metabolite 11-DHC by the enzyme HSD11B1. Analysis of gene expression in different T-cell subpopulations isolated from thymus and spleen showed that *Hsd11b1* is expressed in every T-cell subset (Figure 1c). This suggests that the main source of thymic corticosterone in the mouse, besides *de novo* synthesis in the adrenals, is the metabolite 11-DHC rather than the direct precursor 11-DOC, as we did also fail to detect expression of *Cyp11b1* in mature T cells (Table 1).

A second enzyme, CYP11B2, is able to convert 11-DOC into corticosterone in the adrenal gland so we checked for its expression in thymus and spleen. We could not detect expression of this enzyme either in any of the T-cell subsets analyzed in thymus or spleen, or in non-T cells in thymus (Table 1). Taken together, our expression analysis suggests that none of the cell types we analyzed in the thymus has the capacity to produce GC *de novo* in a cell autonomous manner, a feature shared with mature T cells in the spleen.

### HSD11B1 converts 11-DHC into corticosterone in thymocytes and peripheral T cells and induces cell death

**Figure 2:**
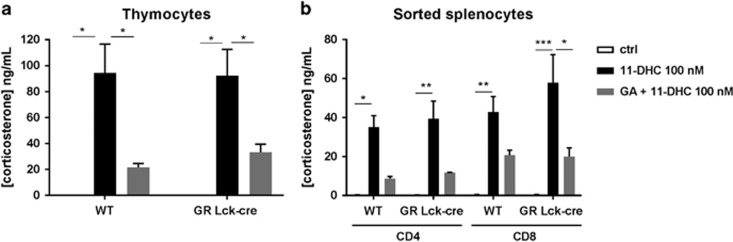
T cells from thymus and spleen are able to transform the inactive metabolite 11-DHC into active corticosterone. Supernatants from overnight cultures of thymocytes (**a**) or sorted splenic CD4+ and CD8+ (**b**) cells either untreated (open bars) or treated with 11-DHC in the absence (black bars) or presence (gray bars) of GA were analyzed for corticosterone content by ELISA. Mean values±S.E.M. from at least three independent experiments are shown

In order to investigate the biological activity of the activating enzyme HSD11B1, we studied the potential of thymocytes and peripheral T cells to transform inactive 11-DHC into corticosterone. To this end, we incubated thymocytes and sorted splenic CD4+ and CD8+ T cells with 11-DHC in the presence or absence of the specific HSD11B1 inhibitor glycyrrhetinic acid (GA), and collected the supernatants for subsequent corticosterone detection. T cells from thymus and spleen showed high capability of producing corticosterone from 11-DHC (Figures 2a and b). Inhibition of HSD11B1 partially blocked 11-DHC conversion in both thymocytes (Figure 2a) and sorted splenocytes (Figure 2b). Basal corticosterone production was very low or undetectable.

**Figure 3:**
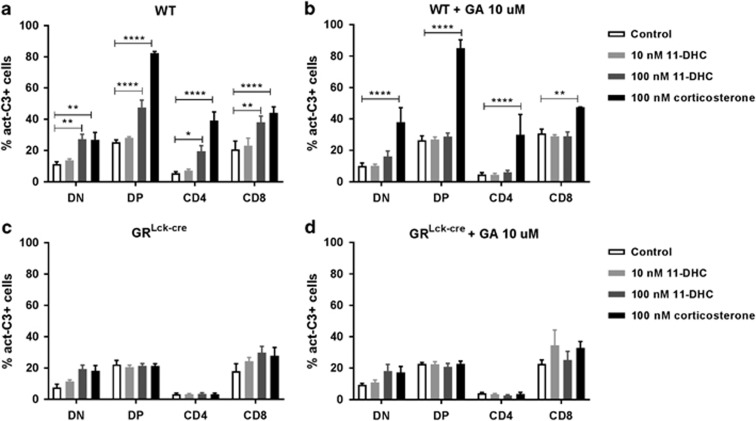
11-DHC induces GR-mediated apoptosis in thymocytes. Total WT or GR-deficient (GR^Lck-cre^) thymocytes were cultured overnight with increasing concentrations of 11-DHC or corticosterone in the presence (**b** and **d**) or absence (**a** and **c**) of GA, and the expression of active caspase-3 was measured by FACS analysis. Mean values±S.E.M. from at least three independent experiments are shown

One of the most prominent effects of GC is to induce apoptosis in developing but also mature lymphocytes.^25, 26, 27, 28, 29^ We hypothesized that if these cells were capable of transforming 11-DHC into its active form, corticosterone, this would bind the GR and trigger cell death. Indeed, 11-DHC administration induced apoptosis in immature DP as well as more mature SP CD4+ and CD8+ thymocytes, as shown by increased levels of activated, that is, cleaved caspase-3 (Figure 3a). Co-incubation with GA completely reversed cell death induced by 11-DHC (Figure 3b). To confirm that the apoptotic effect of 11-DHC was mediated via the GR, we repeated the experiments with GR-deficient thymocytes (GR^Lck-cre^ cells). Importantly, GR^Lck-cre^ thymocytes are not affected by any of the 11-DHC doses or corticosterone itself, strongly supporting the view that apoptosis induced by 11-DHC treatment is due to its transformation into active corticosterone that triggers GR-mediated cell death (Figures 3c and d).

### Lack of evidence for cell autonomous *de novo* GC production in developing thymocytes *in vitro*

**Figure 4:**
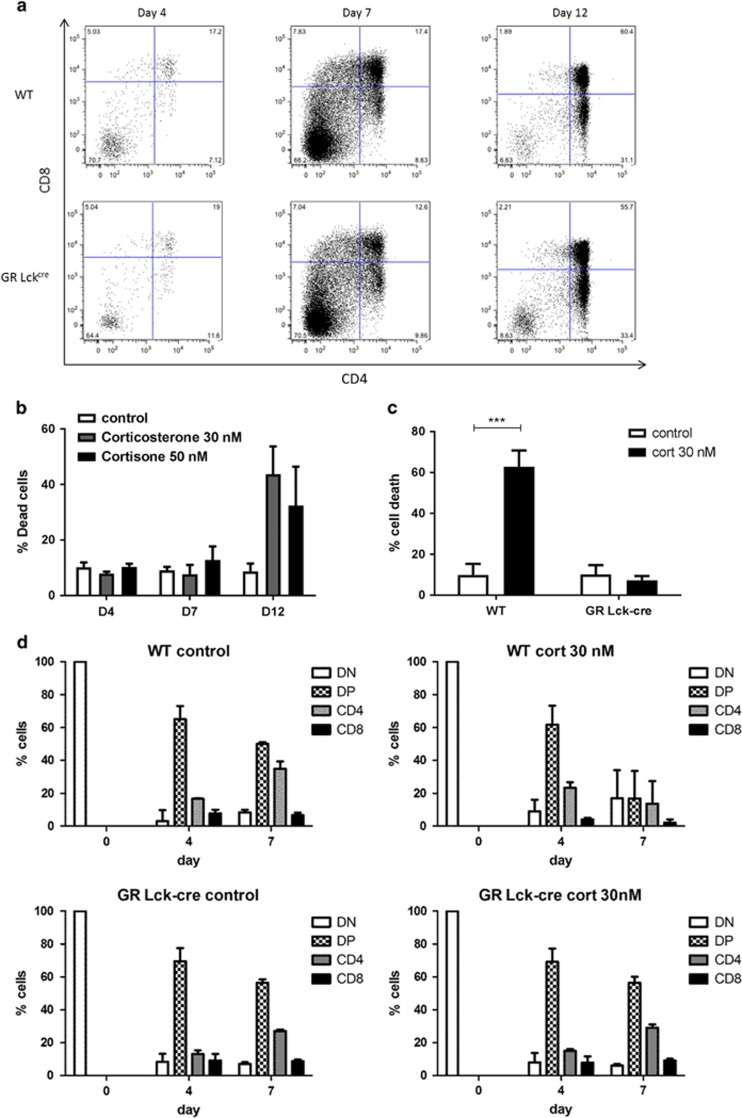
GC treatment affects DP thymocytes in an *in vitro* T-cell development system. (**a**) Early developmental DN1+ 2 (CD44+ CD25+ and CD44+ CD25−) thymocytes from WT and GR^Lck-cre^ thymi were cultured on OP9-DL1 stromal cells and T-cell maturation was assessed during 12 days (**b**) in the presence or absence of corticosterone or cortisone. Cell death was analyzed by Annexin-V/DAPI staining by FACS. (**c**) Immature DN4 (CD44− CD25−) thymocytes were cultured on OP9-DL1 cells for 7 days with or without corticosterone (cort) 30 nM. Cell death was analyzed by Annexin-V/DAPI stainings and (**d**) T-cell subset development was analyzed overtime. Mean values±S.E.M. from at least three independent experiments are shown

Glucocorticoid signaling is suggested to have a crucial role in T-cell development and function, being of special importance for positive selection in the thymus.^9, 10^ Thereby, the GR reportedly associates with the TCR signaling complex^30^ and upon binding of GC, the GR dissociates from this complex, resulting in impaired TCR signaling. However, data on the role of GC on T-cell selection derived from several different *in vivo* animal models carrying GR modifications remain controversial.^31^ Using a mouse strain which lacks the GR specifically in the T-cell lineage (GR^Lck-cre^ mice), we wanted to assess how T cells develop in a well-defined *in vitro* system in the absence of GC signaling. To this end we made use of the OP9-DL1 co-culture system, an *in vitro* model for T-cell development.^32^ Of note, this system is not expected to produce GC *de novo*, as gene expression of the last key enzyme, *Cyp11b1,* was not detectable in our comparative qPCR analysis (Supplementary Table 1). To analyze the development of immature T cells in the absence of GC, we isolated the very early stages of thymocytes (DN1+2) from both wild-type and GR^Lck-cre^ mice and cultured them on OP9-DL1 cells. Development was monitored until the appearance of CD4+CD8low cells, a subset dependent on positive selecting signals provided by the TCR. GR deficiency did not affect thymocyte maturation, according to the subset distribution of CD4+ and CD8+ cells (Figure 4a; see also Figure 4c). Next, we tested whether developing immature DN thymocytes in the OP9-DL1 co-culture system are able to generate active GC from inactive cortisone which would consequently induce cell death. While OP9-DL1 cells expressed detectable amounts of *Hsd11b1*, immature thymocytes expressed much higher levels of this enzyme (Supplementary Table 1). Interestingly, treatment with 50 nM of inactive cortisone produced significant cell death above baseline primarily in DP cells to a similar extent as 30 nM of corticosterone does (Figure 4b; Supplementary Figure 2), indicating conversion of cortisone to active GC. The *Lck* transgene has been reported to become active at the DN3 stage of thymocyte maturation.^33^ We analyzed DN1-4 and DP thymocytes from both wild-type and GR^Lck-cre^ mice for GR protein expression and found partial deletion at the DN4, and complete deletion at the DP stage (Supplementary Figure 3). Thus, the effects of GR deletion in GR^Lck-cre^ cells are expected to impact on cells only at the DP stage or later. Hence, we isolated DN4 thymocytes from wild-type and GR^Lck-cre^ mice and cultured them for 7 days in the absence or presence of corticosterone (30 nM). Cell viability analysis of total thymocytes in culture showed that, as expected, GR-deficient T cells were resistant to corticosterone treatment whereas 60% of WT cells were dead by day 7 of culture (Figure 4c). Subset analysis revealed that DP cells were the main subset affected by corticosterone treatment (Figure 4d). Importantly, if thymocytes would be able to endogenously produce sufficient amounts of *de novo* GC in the OP9-DL1 system under basal conditions, enhanced cellular survival would be expected in cultures with GR-deficient cells, which appeared not to be the case (Figures 4c and d). Our results are consistent with above-mentioned expression analysis of GC-synthesizing enzymes, excluding cell autonomous GC production *de novo* in developing thymocytes. Furthermore, GR signaling induced cell death mainly in DP cells, consistent with published results,^27, 28^ whereas the absence of the GR does not affect T-cell ontology beyond the DN4 stage of thymocyte development.

### Mature T-cell death induced by 11-DHC conversion: effect of TCR activation

**Figure 5:**
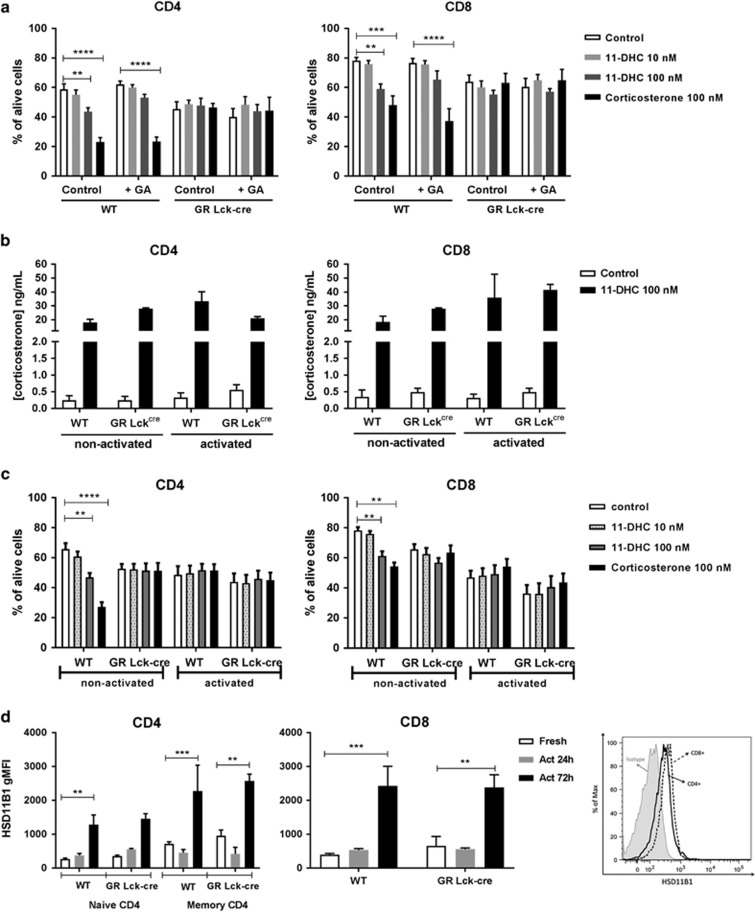
CD4+ and CD8+ splenocytes produce active corticosterone upon 11-DHC administration and its effect is blocked by TCR activation. (**a**) Sorted CD4+ (left panel) and CD8+ (right panel) cells were cultured overnight with increasing concentrations of 11-DHC or corticosterone in the presence or absence (control) of GA, and cell viability was analyzed by Annexin-V/DAPI staining by FACS. Mean values±S.E.M. (*n*=5) are shown. (**b**) Supernatants from resting (non-activated) or activated CD4+ (left panel) or CD8+ (right panel) cells treated overnight with (black bars) or without 11-DHC (open bars) were analyzed for corticosterone content by ELISA. Mean values±S.E.M. (*n*=3) are shown. (**c**) Analysis of GC-induced apoptosis in resting T cells from spleen (‘non-activated’) *versus* TCR-activated CD4+ or CD8+ splenocytes upon treatment with increasing concentrations of 11-DHC or corticosterone (100 nM). (**d**) Total splenocytes from WT or GR-deficient mice were stimulated *in vitro* with soluble anti-CD3 in serum-free medium for 24 or 72 h and HSD11B1 protein was detected in CD4+ (left panel) or CD8+ (middle panel) splenocytes from WT or GR-deficient (GR^Lck-cre^) mice. A representative histogram for HSD11B1 expression for CD4+ and CD8+ cells, including isotype control, is shown in the right panel. Mean MFI values±S.E.M. (*n*=4) are shown

Since both thymocytes and splenic T cells express *Hsd11b1* (Figure 1c) and are able to produce corticosterone from the metabolite 11-DHC (Figures 2a and b), we next incubated sorted splenic CD4+ or CD8+ T cells with 11-DHC to test whether mature T cells are also sensitive to cell death. Similarly to thymic SP CD4+ and CD8+ cells, mature splenic CD4+ and CD8+ cells also underwent cell death upon incubation with the metabolite (Figure 5a) and this effect was partially reversed by the addition of the HSD11B1 inhibitor GA. Experiments performed on GR-deficient T cells showed no sensitivity to either 11-DHC- or corticosterone-induced cell death (Figure 5a).

We next checked whether 11-DHC conversion could be modulated by TCR stimulation as it has been previously suggested.^22^ We activated sorted splenic CD4+ and CD8+ cells with anti-CD3 and anti-CD28 antibodies in the presence of 11-DHC and determined corticosterone production in the supernatants. In agreement with Zhang and colleagues, we observed a slight increase in the conversion rate of 11-DHC compared with resting cells upon activation of both WT CD4+ and CD8+ cells (Figure 5b). GR-deficient CD8+ cells also show higher corticosterone conversion under activation conditions whereas corticosterone levels in GR^Lck-cre^ CD4+ cell cultures was slightly reduced upon activation (Figure 5b).

GC-induced apoptosis in thymocytes and splenic T cells can be blocked via TCR activation.^12, 13, 34^ We determined cell viability from activated CD4+ and CD8+ splenocytes and demonstrated that TCR stimulation blocked CD4+, but not CD8+, cell death induced by corticosterone (Figure 5c). 11-DHC-evoked death was not affected by TCR activation in both CD4+ and CD8+ cells while TCR signaling by itself induced significant cell death, probably mediated by activation-induced cell death.^35^ In order to test whether TCR stimulation modulates HSD11B1 expression, as previously described,^22^ we activated splenocytes with anti-CD3 and monitored the amount of HSD11B1 protein by flow cytometry. The results shown in Figure 5d indicate up-regulation of HSD11B1 in CD4+ (left panel) and CD8+ (right panel) cells derived from WT spleen at 72 h. Similar effects were also observed in GR-deficient CD4+ and CD8+ T cells. Next, we analyzed GR expression levels to verify whether a putative downregulation of the GR could contribute to the resistance to corticosterone after TCR signaling. This appeared, however, not to be the case as GR expression was increased in CD4+ and CD8+ cells alike upon activation (Supplementary Figure 4). These findings clearly show that TCR activation increases HSD11B1 expression and (slightly) enhances enzyme activity in WT CD4+ and CD8+ cells. In addition, reversal of GC-induced death in CD4+ T cells by TCR signaling is not caused by downregulation of GR. Hence, our results point to the existence of a regulatory mechanism in which GC that are intrinsically generated by T cells induce T-cell autonomous death both in thymocytes and mature T cells. Whether cell death induced by regenerated 11-DHC can be prevented by TCR signals *in vivo* remains to be established.

## Discussion

In this study, we addressed the question whether thymocytes and splenic T cells are capable of *de novo* synthesis of GC. Our analysis of gene expression and function of various glucocorticoid metabolic enzymes in both thymocytes and mature T cells strongly suggests that these cells do not produce GC by *de novo* synthesis but rather convert inactive metabolites. T cells that produce GC in this manner undergo GR-mediated autonomous cell death under basal conditions, but may become resistant to GC upon TCR-induced activation.

The major production sites of GC are the adrenal glands that release these hormones in a basal, circadian manner and upon mental, physical or immunological stress.^1, 36^ However, local production of GC by the immune system,^10, 14, 15, 17, 37^ has been shown both *in vitro* and *in vivo* in a variety of experimental systems.^10, 20, 21, 38, 39^ At the functional level, Ashwell and colleagues proposed that GC antagonize TCR signaling (and *vice versa*), thereby contributing to positive selection of immunocompetent T cells.^7, 40^ In the thymus, TEC cells reportedly produce *de novo* GC probably affecting thymocytes in a paracrine manner but whether thymocytes themselves also produce *de novo* GC is, however, controversial.^17, 20, 23^ Our data support the notion that conversion by thymocytes and T cells of inactive metabolites to GC is substantial (Figures 2a and b) and functionally relevant as it produces cell death in the same cells (Figures 3a–d; Figure 5a) while we find no evidence for competence for *de novo* GC synthesis. Moreover, our observation that immature DN thymocytes differentiating in the OP9-DL1 co-culture system into DP cells also display substantial cell death upon addition of an inactive metabolite corroborates this view. These results show that immature thymocytes actively generate GC from inactive metabolites that may induce cell death if not counteracted by TCR signaling of the appropriate signaling strength.

The question whether the effects of newly generated GC are indeed mediated via the GR is important since GC have been shown to bind receptors other than the GR.^41^ The mineralocorticoid receptor (MR) that displays high affinity for corticosterone can mediate effects on T-cell function opposite to that of the GR.^42^ However, our data show that T-cell death induced by T-cell derived corticosterone is mediated exclusively by the GR as cell death was completely prevented in GR-deficient T cells (Figures 3c and 5a).

The effects of 11-DHC on thymocyte and T-cell cell death we observed occurred at physiological concentrations of 11-DHC. Importantly, as free corticosterone levels are only comparable to 11-DHC during the diurnal peak or stress response, 11-DHC is the main bioavailable substrate that can be converted to active GC.^43, 44^ Therefore, we assume that conversion of 11-DHC contributes a considerable proportion of intracellular active GC. However, animal models lacking HSD11B1 or CYP11A1/B1 specifically in T cells or TEC are needed to elucidate the functional relevance of the conversion of GC metabolites or the quantitative contribution of *de novo* synthesized GC, respectively.

CYP11A1 converts cholesterol to pregnenolone that serves as the precursor for all other steroids. Our findings indicate that only immature thymocytes express low levels of the *Cyp11a1* gene which is lost at the mature SP stage. A previous study reported *Cyp11a1* expression at a later stage of thymocyte development in newborn mice.^21^ The reason for this discrepancy is not known although the age of the animals has indeed been shown to affect both expression levels and activity of CYP11A1 and other GC metabolic enzymes.^10, 20, 45^ In contrast to CD4+ SP thymocytes, low levels of *Cyp11a1* were detected in thymic CD4+Foxp3+ SP cells, but whether this also translates in significant protein expression levels and production of pregnenolone remains to be established. In an attempt to enhance potential *de novo* GC production we incubated both thymocytes and purified splenic CD4+ and CD8+ cells with the CYP11A1 substrate 22R-hydroxycholesterol. However, we found that this compound produced significant cell death that appeared to be independent of the GR (Supplementary Figure 5).

While splenic naive CD4+ and CD8+ T cells do not express *Cyp11a1*, upon TCR activation it becomes detectable (Supplementary Figure 6a), in agreement with Mahata *et al.*^46^ who reported *Cyp11a1* expression after activation selectively in Th2, but not naive, T cells in a helminth infection model. Interestingly, these authors reported pregnenolone production but did not detect expression of enzymes (HSD3B1, CYP17A1), or production of steroids (progesterone, 17-hydroxypregnenolone), further downstream the GC synthesis pathway. Pregnenolone itself induced immunosuppressive effects on the Th2 response, suggesting no further metabolic processing is needed. Recently, CYP11A1 reportedly regulates effector CD8+ T-cell conversion.^47^ In any case, it is currently unknown how pregnenolone mediates its effects in CD8+ cells. We hypothesize that pregnenolone may serve an alternative role in immune regulation, rather than being a precursor of the GC synthesis pathway.

TCR activation reportedly upregulates HSD11B1 expression and activity.^22^ We confirmed increased expression of HSD11B1 at mRNA (Supplementary Figure 6b) and protein levels (Figure 5d) in both CD4+ and CD8+ T cells after activation and enzyme activity appeared slightly increased in activated T cells. As TCR signaling induces the production of pro-survival cytokines and their receptors, we assume that these or other TCR-induced mechanisms counteract the apoptotic effects of active GC like corticosterone.^48, 49, 50, 51^

Our observation that activation by the TCR protects CD4+ T cells from GC that induce cell death may have important consequences for T-cell selection and peripheral T-cell homeostasis. In the thymus, only those thymocytes that express TCRs capable of signaling can undergo positive selection and this may protect from GC-induced cell death. Indeed, activation of both thymocytes and mature splenic T cells via their TCRs has been shown to protect against cell death induced by exogenous GC.^13^ Thymocyte production of GC by endogenous conversion of metabolites may represent a mechanism of autonomous cell death control independently of the circadian, fluctuating concentrations of adrenal-derived GC. This would enable thymocytes to undergo positive selection in the presence of basal, less variable levels of GC. In such a scenario, escape from autonomously produced, GC-evoked cell death may be necessary for immature thymocyte positive selection. In the periphery, T-cell homeostasis is regulated by GR expression levels as transgenic mice expressing increased GR levels specifically in T cells display reduced T-cell numbers.^16^ Our findings indicate that mature T cells similarly require TCR signaling to avoid cell death by exogenous GC. Whether the same holds true *in vivo* for endogenous GC that are either cell-intrinsically regenerated and/or adrenal-derived remains to be established.

The clinical relevance of our findings is shown in different animal experimental models of inflammation where HSD11B1 deficient mice show an exacerbation of inflammatory symptoms and impaired resolution.^52^ Thus, the presence of functional HSD11B1 enables T cells to generate GC autonomously and provides them with an intrinsic means to control T-cell development, selection and function.

## Materials and methods

### Mice

GR^flox^ mice^53^ were bred on a C57BL/6 background to mice expressing Cre as a transgene under the control of the proximal *Lck* promoter (LckCre) as described.^54^ They were housed in the Central Laboratory Animal Facilities of the Medical University of Innsbruck under standard light cycles and temperatures, and food and tap water were available *ad libitum*. Mice at 5–12 weeks of age were sacrificed by cervical dislocation and organs were extracted for analysis. Lck-cre^−^ (WT) mice were used as littermate controls for GR^Lck-cre^ mice.

### Flow cytometry

Cell suspensions were prepared in KDS-BSS buffer containing 10% FCS. Cells were stained with combinations of the following antibodies for 20 min at 4 °C: anti-CD4-PerCP (clone RM4-5) (eBiosciences, San Diego, CA, USA), anti-CD8-PECy7 (clone 53-6.7), anti-B220-APC/Cy7 (clone RA3-6B2) (both from Biolegend, San Diego, CA, USA). DAPI and Annexin-V-AF647 (eBiosciences) were used to quantify or gate out apoptotic or dead cells.

For CD4-CD8- (DN) thymocyte subset isolation, we performed MACS purification with Sheep anti-rat IgG Dynabeads (Invitrogen, Eugene, OR, USA) using purified anti-CD4 (clone GK1.5) and anti-CD8 (clone 53-58) antibodies (Biolegend). Subsequently, the cells were stained with anti-CD25-PE (clone 3C7), anti-CD8-PerCP (clone 53-6.7) (Biolegend), anti-CD44-APC (clone IM7), anti-TCR*β*-FITC (H57-597) and anti-CD69-eF450 (clone H1.2F3) (eBiosciences); cells to be excluded were stained with biotinylated antibodies (anti-TCR*γδ* (clone eBioGL3) (eBiosciences), anti-B220 (clone RA3-6B2), anti-Gr1 (clone RB6-8C5), anti-Ter119 (Ter119), anti-Mac1 (clone M1/70) and anti-NK1.1 (PK136)), followed by Streptavidin-PECy7 (Biolegend). DN subpopulations were identified as CD44+ CD25− (DN1), CD44+ CD25+ (DN2), CD44− CD25+ (DN3) and CD44−CD25− (DN4).

For intracellular staining we used BD Cytofix and BD Cytoperm reagents (BD Pharmingen, San Jose, CA, USA) and stained with anti-GR (clone D6H2L) (Cell Signaling, Danvers, MA, USA), anti-HSD11B1 (clone EP9406) (Abcam, Cambridge, UK) or Anti-rabbit (clone DA1E) isotype control (Cell Signaling), followed by a secondary antibody (goat anti-rabbit IgG AF647 (Invitrogen)).

For detection of Active Caspase-3 we first stained 1 × 10^6^ cells for surface markers and then proceeded with fixation and permeabilization using the PE-active Caspase-3 apoptosis kit (BD Pharmingen) according to the manufacturer’s instructions.

### Cell culture

Single-cell suspensions were prepared from thymi or spleens of WT and GR^Lck-cre^ mice. For T-cell development experiments, 6 × 10^3^ cells/ml DN1+2 or DN4 cells from thymus were cultured in a 24-well plate containing OP9-DL1 cells as described.^55^ In some experiments, cells were treated with 30 nM corticosterone (Sigma, St. Louis, MO, USA) from day 0. Treatment with corticosterone was refreshed when cells were transferred into a new OP9-DL1 plate (every third day). Cells were able to differentiate to a similar extent than they do in RPMI supplemented with 10% FCS (data not shown).

For TCR stimulation of total splenocytes 1 × 10^6^cells/ml were seeded in a 96-well plate in X-vivo 20 (Lonza, CH) serum-free medium (supplemented with 50 *μ*M *β*-Mercaptoethanol, 100 U/ml Penicillin/Streptomycin, 2 mM l-Glutamine, 1 mM Na-Pyruvate and non-essential amino acids) and treated with soluble anti-CD3 (Biolegend) 1 *μ*g/ml for 24 or 72 h.

Sorted CD4+ or CD8+ cells (2,5 × 10^5^ cells/ml) were seeded in the same conditions in anti-CD3 coated wells (5 *μ*g/ml) and treated with soluble anti-CD28 (1 *μ*g/ml; both antibodies from Biolegend) in the presence of 100 U/ml IL-2 (PreproTech, Rocky Hill, NJ, USA). Cells were incubated overnight either with 11-DHC (MyBioSource, San Diego, CA, USA) at 10 or 100 nM, corticosterone (100 nM) or staurosporine (100 nM) as a control for apoptosis, in the presence or absence of 10 *μ*M glycyrrhetinic acid (GA) (Sigma).

To study effects of 11-DHC on thymocytes, 1.5 × 10^6^ cells/ml total thymocytes were seeded in 96-well plates in X-vivo 20 medium and treated overnight as described above for splenic CD4+ and CD8+ cells.

### Quantitative real-time PCR

Total RNA was isolated from 1 × 10^5^ cells using Quick-RNA MicroPrep kit (Zymo Research, Irvine, CA, USA) and cDNA was synthesized using iScript cDNA Synthesis Kit (BioRad, CA, USA), according to the manufacturer’s instructions. Real-time PCR was done using the following primers for SYBRGreen: *Cyp11a1* forward primer 5-GAC CTG GAA CCA TGC A-3 and reverse primer 5-TGG GTG TAC TCA TCA GCT TTA TTG A-3; *Actin-beta* forward primer 5-ACT GGG ACG ACA TGG AGA AG-3 and reverse primer 5-GGGGTG TTG AAG GTC TCA AA-3; *Keratin-8* forward primer 5-CTC CGG CAGATC CAT GAA GA-3 and reverse primer 5-GCT CGG CTG CGA TTG G-3. Some primer pairs were TaqMan Assays from Applied Biosystems (Thermo Fischer Scientific, Waltham, MA, USA): *Cyp11b1* (Mm01204952_m1), *Hsd11b1* (Mm00476182_m1), *Cyp11b2* (Mm00515624_m1), and *Actin-beta* (Mm00607939_s1). Quantitative RT-PCR was performed using the StepOnePlus system (Applied Biosystems, Thermo Fischer Scientific) and DyNAMO Flash SYBR master mix (Finnzymes, Thermo Fischer Scientific) or Luminaris Color Probe (Thermo Fischer Scientific) for TaqMan gene expression assays according to the manufacturer’s instructions. The results were normalized to *Actin-beta* expression and evaluated using the ΔΔCt relative quantification method.

### ELISA for corticosterone

Supernatants from CD4+ and CD8+ splenocytes and total thymocytes that were treated overnight with 11-DHC in the presence or absence of 10 *μ*M GA were analyzed for corticosterone content by ELISA (Enzo Life Sciences, CH) according to the manufacturer’s instructions. Complete serum-free culture medium did not contain detectable amounts of GC.

### Statistics

Estimation of statistical differences between groups was carried out using the unpaired Student’s *t*-test or two-way ANOVA test, where appropriate. Asterisks (*, **, ***, ****) indicate statistical significance (*P*<0.05; *P*<0.01; *P*<0.001; *P*<0.0001, respectively).

## Figures and Tables

**Table 1:** Expression of GC metabolic enzymes (ΔCt) in different cell types from 5 to 12 weeks old mice

Δ**Ct**	***Cyp11a1***		***Cyp11b1***		***Cyp11b2***		***Hsd11b1***		***n***
	**Mean**	**S.E.M.**	**Mean**	**S.E.M.**	**Mean**	**S.E.M.**	**Mean**	**S.E.M.**	
Adrenal	0.5	0.4	0.4	0.3	2.4	0.4	4.0	0.2	*n*≥4
Liver	11.8	0.3	N.d.		N.d.		0.7	0.1	*n*≥4

*T-cell developmental stages*									
DN1+2	14.7	0.4	N.d.		N.d.		7.0	0.1	*n*=6
DN3	13.8	0.3	N.d.		N.d.		6.3	0.2	*n*=6
DN4	16.4	0.4	N.d.		N.d.		9.9	0.0	*n*=6
DP-TCRlow	10.9	0.7	N.d.		N.d.		6.3	0.4	*n*≥3
DP-CD69+	14.1	0.7	N.d.		N.d.		7.3	0.8	*n*≥3
DP-TCRhigh	11.1	0.7	N.d.		N.d.		5.9	0.3	*n*≥3
T-CD4	N.d.		N.d.		N.d.		6.9	1.0	*n*≥3
T-Tregs	11.0	0.3	N.d.		N.d.		7.1	0.3	*n*≥3
T-CD8	N.d.		N.d.		N.d.		6.6	0.1	*n*≥3
S-Naive	N.d.		N.d.		N.d.		6.2	0.3	*n*≥3
S-Tregs	N.d.		N.d.		N.d.		5.8	0.6	*n*≥3
S-CD8	N.d.		N.d.		N.d.		2.5	0.7	*n*≥3

*Non-T cells*									
TST	9.2	1.0	N.d.		N.d.		6.4	0.3	*n*=8
Macrophages	N.d.	N.d.	N.d.		N.d.		10.0	0.1	*n*=3
DCs	11.5	0.4	N.d.		N.d.		9.5	0.1	*n*=3
B cells	11.9	1.0	N.d.		N.d.		10.9	0.1	*n*=3

Abbreviations: DCs, dendritic cells; N.d., not detectable; S, spleen; T, thymus; TST, enriched thymic stromal tissue

Mean values±S.E.M. for ΔCt (referred to *Actin* expression) from, at least, three independent experiments are shown
