# Supplementary material for: T-cell autonomous death induced by regeneration of inert glucocorticoid metabolites
Source: Cell Death Dis. 2017 Jul 20;8(7):e2948–. doi: 10.1038/cddis.2017.344 (PMC5550885; doi:10.1038/cddis.2017.344)
Supplement: Supplementary Information [file cddis2017344x1.docx]

**Supplementary Material Legends**

Figure S1) Thymic stromal tissue (TST) is TEC-enriched. Real time qPCR analysis of *Krt-8* expression (expressed as 2^-ΔΔCt^) was analyzed in total thymus samples compared to enriched TST samples. Mean values ± SEM from three independent samples are shown.

Suppl. Table 1: Expression of GC metabolic enzymes (ΔCt) in OP9-DL1 cells compared to immature thymocyte subsets from 5 to 12 weeks old mice. Mean values ± SEM for ΔCt (referred to *Actin* expression) from, at least, three independent experiments are shown. n.d.= not detectable

Figure S2) Inactive GC metabolites affect immature thymocyte development. Immature DN 1+2 cells (CD44+ CD25- and CD44+ CD25+) were seeded on OP9-DL1 cells and their development monitored over 12 days in the presence or absence of corticosterone or cortisone. Thymocyte subset distribution at day 7 of culture is depicted. Mean values ± SEM of three independent experiments are shown.

Figure S3) GR deletion in GR^Lck-cre^ immature thymocytes becomes detectable at the DN4 (CD44- CD25-) stage. Representative histograms of GR expression at different T-cell developmental stages (DN2, DN4 and DP) for WT and GR^Lck-cre^ thymocytes are shown.

Figure S4) GR expression in CD4+ (left panel) or CD8+ (right panel) splenocytes from WT or GR-deficient (GR^Lck-cre^) mice upon *in vitro* TCR activation of total splenocytes. Mean MFI values ± SEM from three independent experiments are shown.

Figure S5) T-cells do not convert the CYP11A1 substrate 22R-Hydroxycholesterol into corticosterone: a) Total thymocytes of WT (left panel) or GR^Lck-cre^ (right panel) were cultured overnight in the presence or absence of 22R-Hydroxycholesterol or corticosterone and the expression of active caspase-3 was measured by FACS analysis. b) Sorted CD4+ and CD8+ cells from WT and GR^Lck-cre^ mice were treated overnight with 22R-Hydroxycholesterol or corticosterone and cell viability was analyzed by Annexin-V/DAPI staining. Mean MFI values ± SEM from three independent experiments are shown.

Figure S6) Activated splenic CD4+ and CD8+ cells up-regulate *Cyp11a1* and *Hsd11b1* mRNA expression. Sorted CD4+ and CD8+ cells were *in vitro* stimulated with anti-CD3 and anti-CD28 and *Cyp11a1* (a) or *Hsd11b1* (b) expression was analyzed after 24 and 72h of activation. Mean values (2^-ΔΔCt^) ± SEM from 3 independent experiments are shown.
