# Supplementary figures and images for: T-cell autonomous death induced by regeneration of inert glucocorticoid metabolites
Source: Cell Death Dis. 2017 Jul 20;8(7):e2948–. doi: 10.1038/cddis.2017.344 (PMC5550885; doi:10.1038/cddis.2017.344)

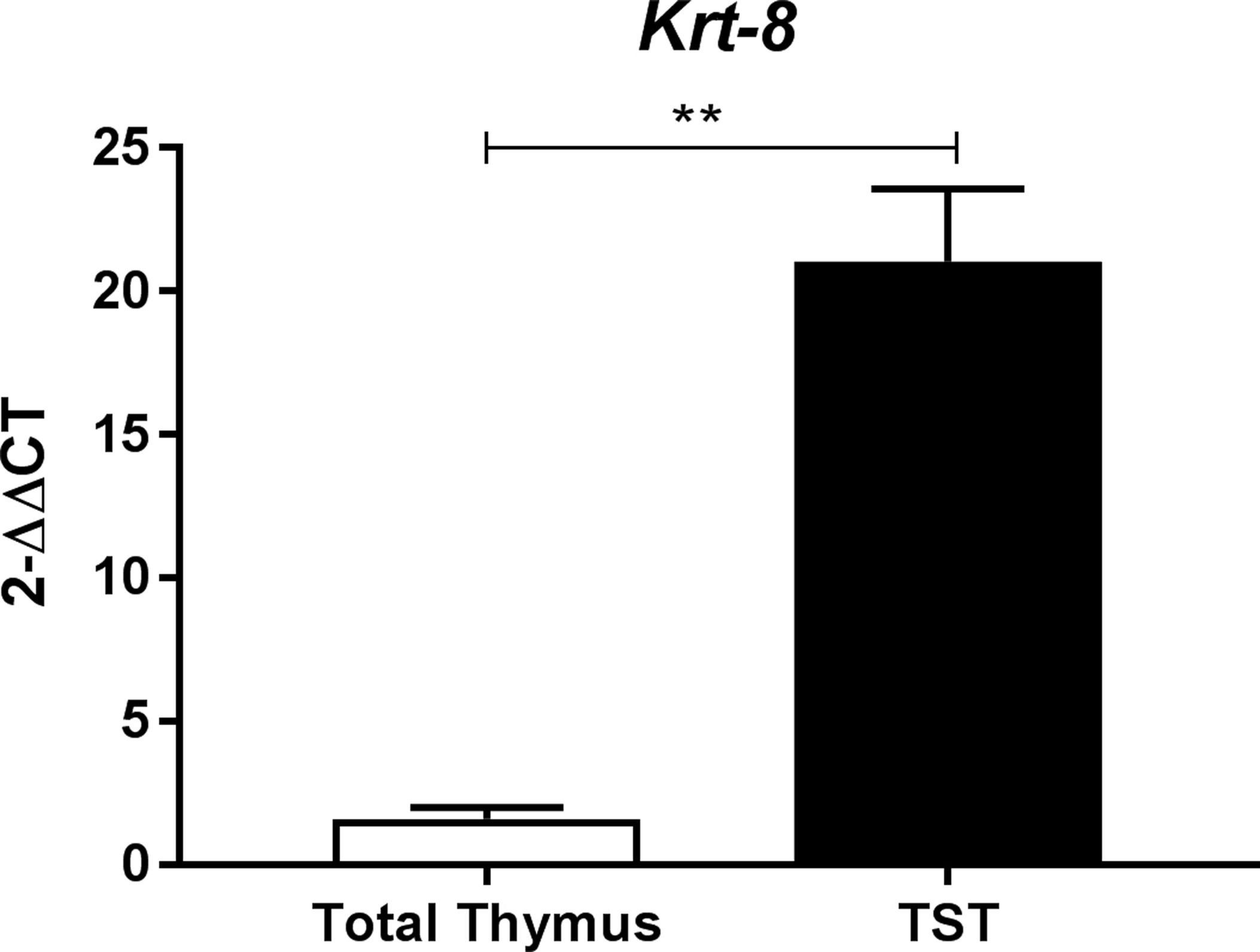

Supplement: Supplementary Figure 1 [file cddis2017344x2.tif]

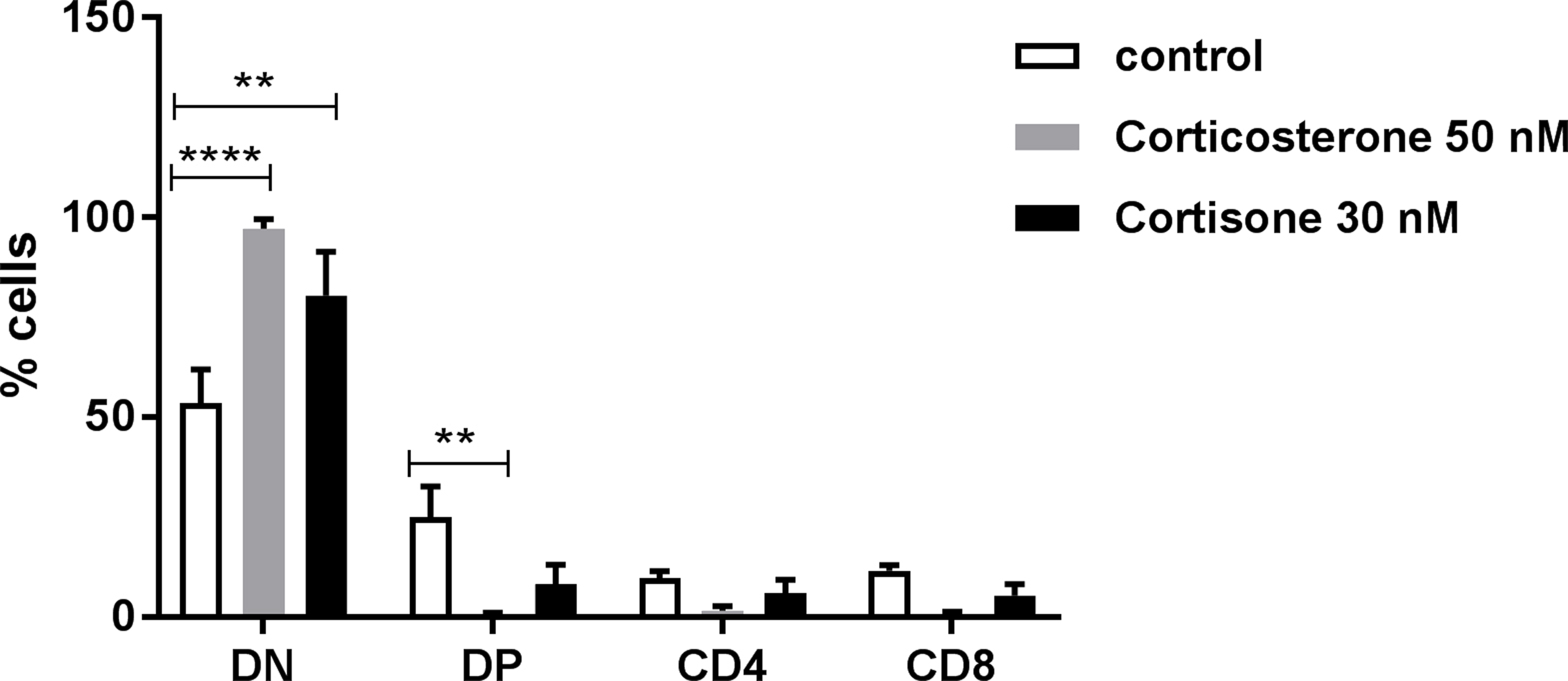

Supplement: Supplementary Figure 2 [file cddis2017344x4.tif]

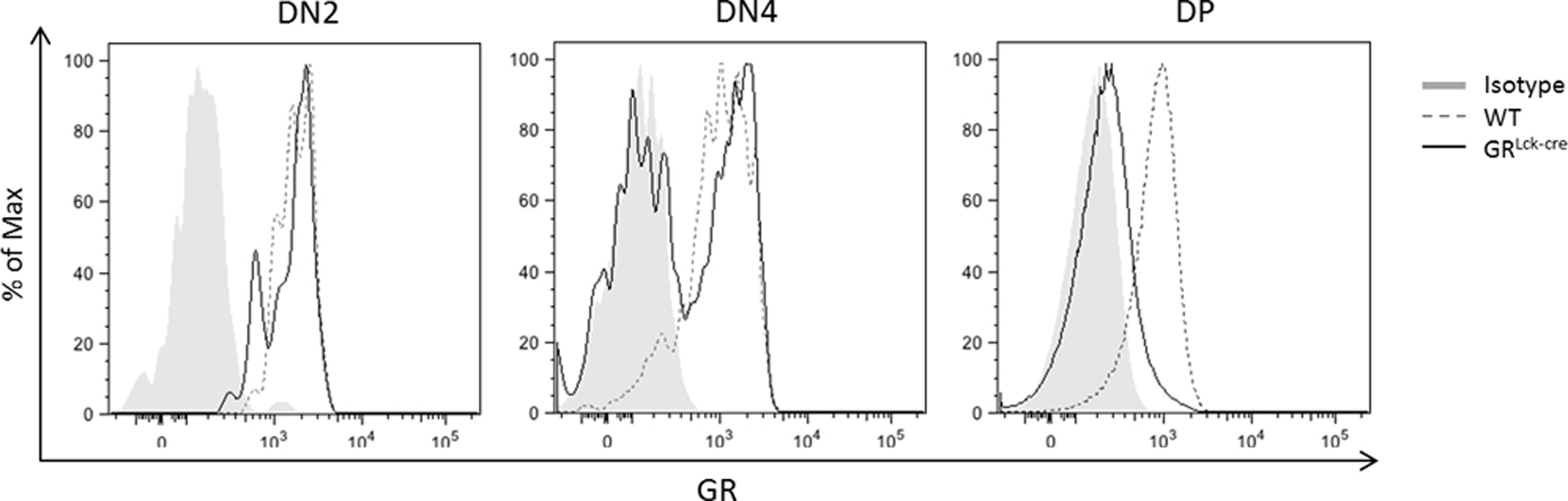

Supplement: Supplementary Figure 3 [file cddis2017344x5.tif]

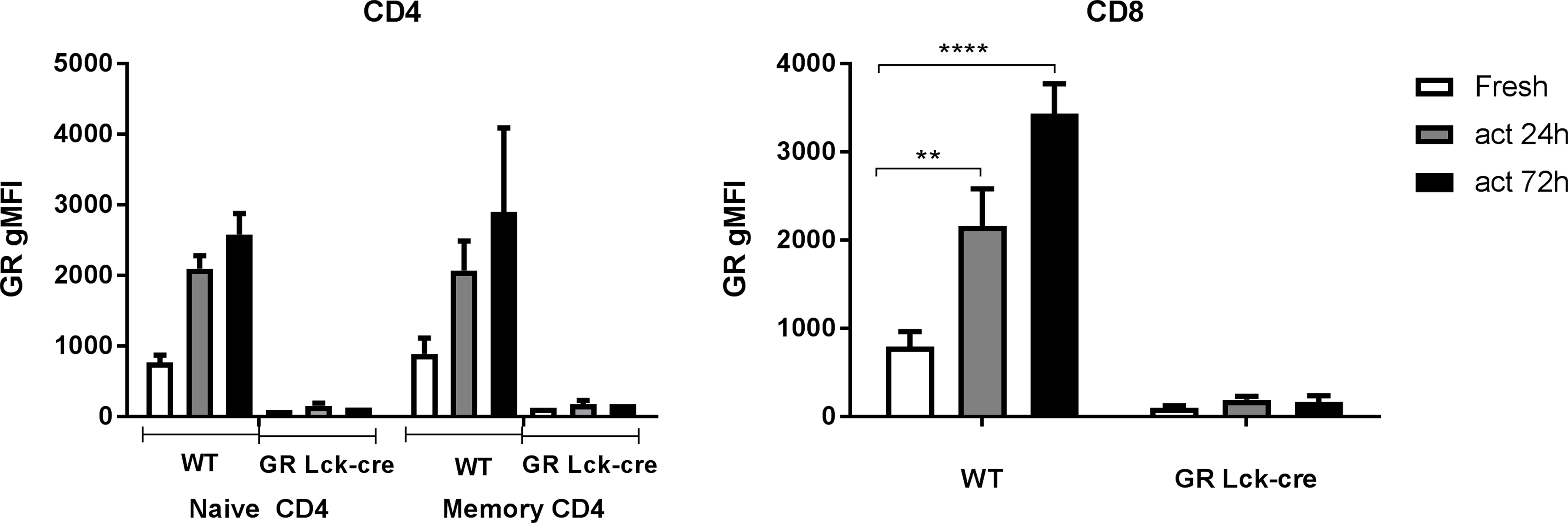

Supplement: Supplementary Figure 4 [file cddis2017344x6.tif]

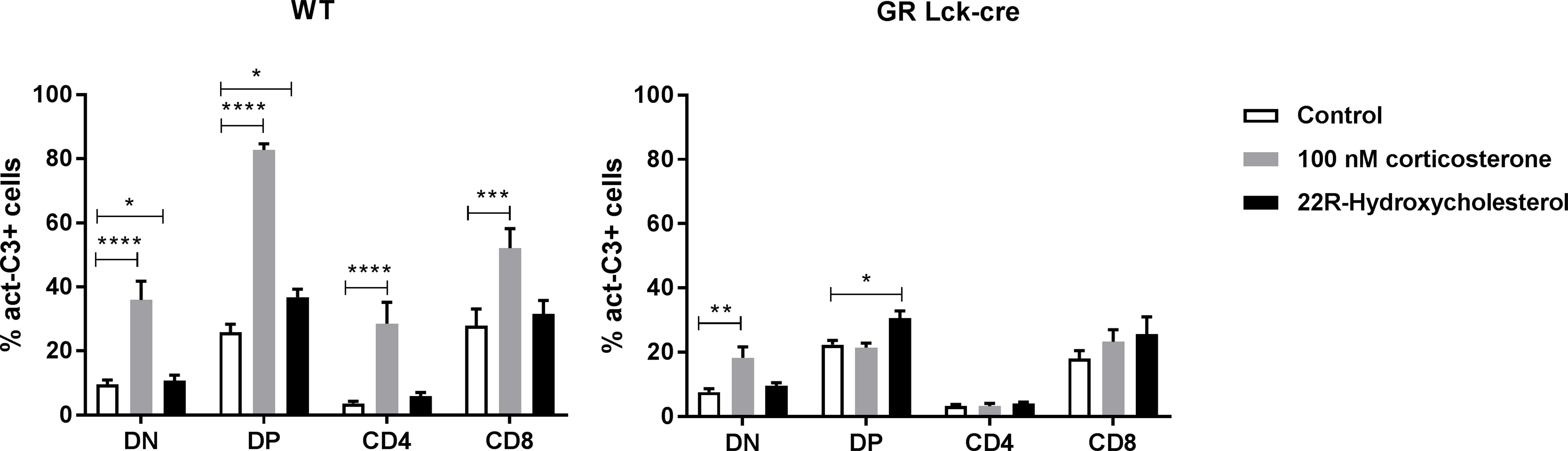

Supplement: Supplementary Figure 5 [file cddis2017344x7.tif]

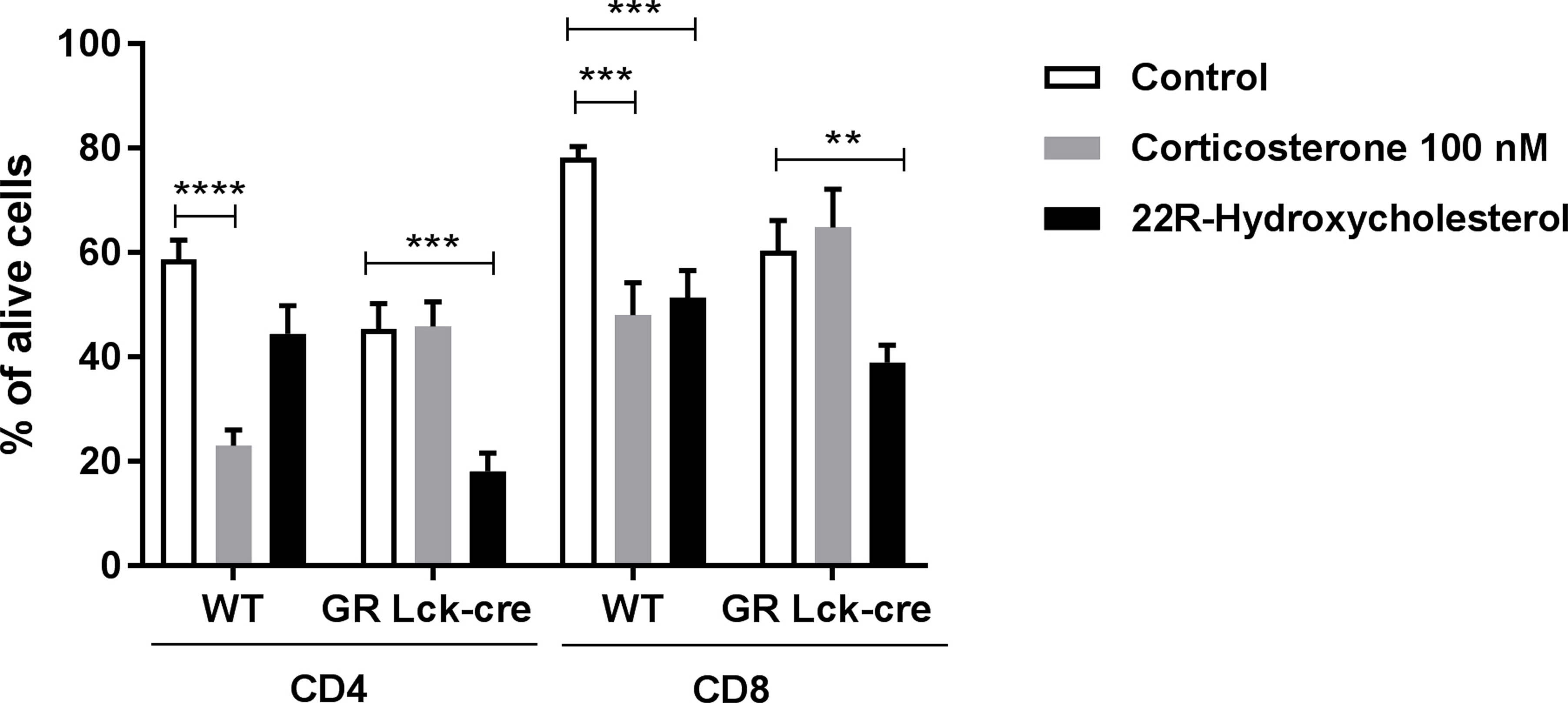

Supplement: Supplementary Figure 6 [file cddis2017344x8.tif]

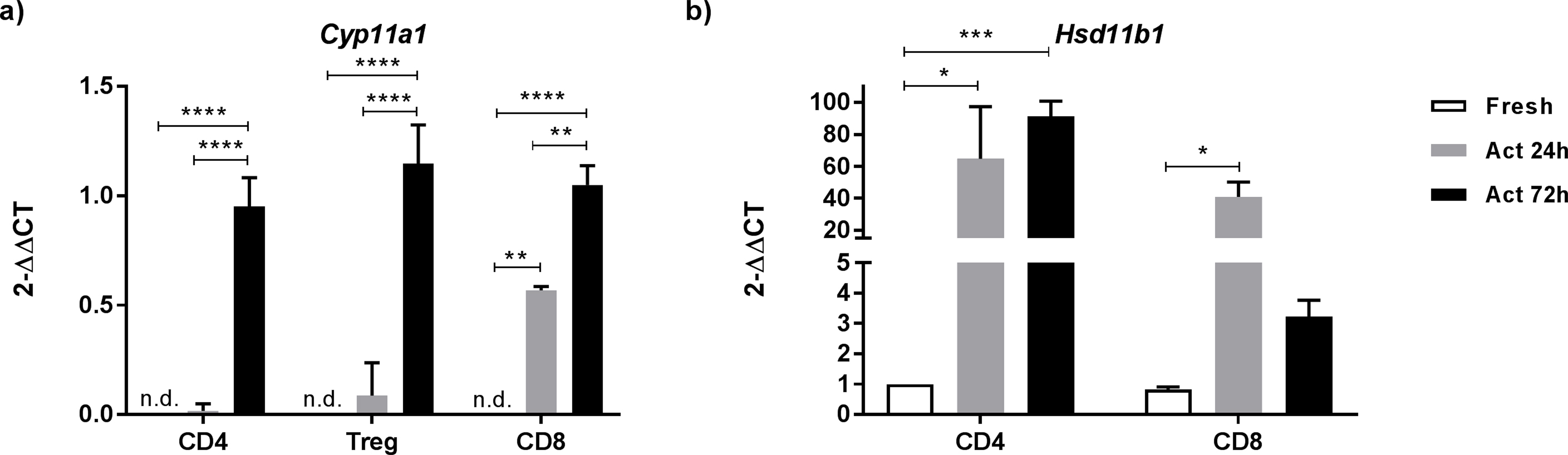

Supplement: Supplementary Figure 7 [file cddis2017344x9.tif]
